# Study on the policy implementation of the Guangdong-Hong Kong-Macao joint graduate training program and regional talent development

**DOI:** 10.1371/journal.pone.0338940

**Published:** 2025-12-29

**Authors:** Sirui Li, Johnny Fat Iam Lam

**Affiliations:** 1 Faculty of Humanities and Social Sciences, Macao Polytechnic University, Macao, China; 2 Faculty of Humanities and Social Sciences, Macao Polytechnic University, Macao, China; Yunnan University, CHINA

## Abstract

To address the existing literature’s neglect of the micro-mechanisms involved in implementing joint postgraduate training policies in the Guangdong–Hong Kong–Macao Greater Bay Area, this study applies resource dependence theory to examine the logic of organisational interactions and their impact on talent development. Based on an analysis of 47 joint cultivation projects through qualitative case studies, the study identifies symbiotic, dominant, and competitive dependency relationships among cooperative entities, shaped by differences in resource endowments, which profoundly influence the stability of cooperation models. Policy effectiveness is primarily achieved through two intermediary mechanisms: “resource integration,” which consolidates financial, human, and knowledge resources, and “collaborative governance,” which builds an institutionalised and organised collaborative network. Significant differences exist between “university–university” and “university–institute” models in terms of both resource integration and governance effectiveness. Policy implementation enhances the quality and scale of talent cultivation while simultaneously promoting the development of the regional innovation ecosystem. The findings indicate that the effectiveness of joint cultivation hinges on micro-governance grounded in resource dependence at the implementation level. Future policy optimisation should prioritise balanced resource allocation and the institutionalisation and standardisation of cooperation models, thereby facilitating the transformation of regional talent governance from a “policy-driven” to a “system-driven” approach.

## 1. Introduction

Variations in regional economic development often arise from differences in the concentration of intellectual capital and the efficiency of its mobility. This process depends heavily on cross-border talent mobility mechanisms within an institutionalised openness framework [1]. As a national strategic city cluster, the Greater Bay Area requires specialised talent to drive industrial upgrading and technological breakthroughs, with higher education synergy serving as a core pathway for building a regional innovation ecosystem. Recent practice demonstrates that the Greater Bay Area has consistently dismantled barriers to talent mobility through intensive higher education collaboration initiatives—such as enrolling over 1,500 participants in joint postgraduate programs by 2023, attracting more than 120,000 Hong Kong and Macao students to study in Guangdong, and establishing over 60 inter-university cooperation platforms—thereby injecting new endogenous momentum into regional economic growth [2].

However, existing studies on joint training mechanisms in the Greater Bay Area exhibit significant limitations. While prior research has addressed policy design, macro-level synergy, governance efficiency, and resource dependence, a key gap is the lack of in-depth examination at the micro level of policy implementation—particularly studies applying resource dependence theory to systematically analyze the logic of resource exchanges, dependency structures, and their role in shaping cooperation mechanisms among local governments, universities, and other organizations in Guangdong–Hong Kong–Macao joint training, as well as their ultimate impact on regional talent cultivation effectiveness. Most existing studies focus on macro-level policy design [3], management processes [4,5], and governance efficiency assessments [6], or briefly address resource dependence without systematically testing, either theoretically or empirically, the dynamic linkages between its type, intensity, and implementation outcomes.

To address this research gap, the paper poses the following core question: How does the Guangdong–Hong Kong–Macao Greater Bay Area’s joint training policy form a stable implementation network through resource complementarity, power negotiation, and cooperative rules among organisations, and how does this network contribute to building regional talent capacity? The study aims to construct an integrative analytical framework grounded in resource dependence theory, focusing on the Greater Bay Area’s joint cultivation program to examine the micro-level interactions among multiple actors during policy implementation and their impact on talent development. This paper’s core contributions are threefold, (1) Theoretical integration, deeply combining resource dependence theory with regional policy implementation mechanisms and proposing a three-dimensional analytical framework of “resource–structure–mechanism,” thereby extending the theory’s application boundaries in regional policy research; (2) Methodological innovation, drawing on 47 joint cultivation project cases to categorize and summarize types of resource dependence and integrate them into the analysis framework; and (3) Object focus, systematically examining the Guangdong–Hong Kong–Macao joint cultivation program to address the gap in research on the micro-mechanisms linking regional policy and talent development. By constructing a theoretical framework and conducting cross-case comparative analysis, the paper deepens the understanding of the implementation logic of the Greater Bay Area’s joint training policy. It clarifies how variations in resource allocation affect the effectiveness of cooperation mechanisms and the performance of talent. The paper is structured as follows: Section 1 reviews the relevant literature; Section 2 constructs the theoretical framework; Section 3 introduces the research methodology and data; Section 4 presents findings and discussion; and Section 5 concludes the study.

## 2. Literature review

Research by domestic and international scholars on regional talent development and education policy can be grouped into three main thematic areas. The first concerns the synergies between regional economic development and talent. Florida’s (2022) creative class theory underscores the central role of talent concentration in regional innovation [7]; the EU’s Erasmus Program demonstrates that cross-border educational collaboration enhances the efficiency of human resource integration [8]; and Qiu and Osman (2024) examine the coupled and coordinated development of regional innovation and talent employment in Malaysian and Chinese cities [9]. In China, research has examined regional synergistic practices in the Beijing–Tianjin–Hebei and Yangtze River Delta regions, finding that talent allocation efficiency and regional innovation capacity can be significantly enhanced through policy guidance, platform integration, and institutional alignment [10,11].

The second thematic area concerns institutional adaptation in higher education collaboration. International studies indicate that dual-tutorship arrangements can enhance graduate students’ academic competence [12], yet institutional differences often create barriers to implementation. In China, Zhang and Chen (2023) used the University of Applied Sciences as a case study to explore improving graduate education quality through deep university–industry cooperation [13]. Similarly, initiatives such as the “China–Africa University Cooperation Program” and the “China–Russia Engineering Education Alliance” provide empirical support for cross-regional collaboration [14]. Recent studies have also explored university–enterprise collaboration: for instance, the “Three Links and Five Harmonies” model optimises talent cultivation through enterprise feedback on curriculum design [15], and joint university–enterprise training enhances the practical skills of engineering graduate students [16].

The third thematic area examines the relationship between education policy implementation and resource dependence. Pfeffer and Salancik’s (2003) resource dependence theory provides a foundation for understanding multi-agency policy collaboration, while Fowles (2013) and Powell and Rey (2015) further analyze the dependency structures of higher education organizations in acquiring financial resources [17,18]. Domestic scholars have examined the interaction structures and coordination mechanisms among the three regions in policy implementation within the context of Guangdong–Hong Kong–Macao cooperation [19,20], and have attempted to develop quantitative evaluation models to assess policy implementation effectiveness [21]. However, most of these studies focus on macro-level interaction structures and coordination mechanisms, without delving into the specific types and dynamics of inter-organisational resource exchanges.

While these studies lay an important foundation for understanding regional education synergies, they collectively highlight a critical research gap. Much of the literature on joint education in the Greater Bay Area analyses policy frameworks and governance structures at the macro level but fails to open the “black box” of policy implementation [22,23]. Although some studies have employed the perspective of resource dependence theory, their analyses often lack systematic depth and fail to unpack the specific mechanisms through which resource asymmetry influences organizational behavior and policy adaptation [19,24]. They fail to systematically address the following core questions: What key resources (e.g., policy indicators, faculty, research platforms) do different actors (e.g., local governments and universities) rely on within specific policy implementation networks? How does the asymmetric distribution of these resources shape inter-organisational dependencies (e.g., symbiosis, dominance, or competition)? Moreover, how do these micro-dependencies ultimately determine the effectiveness of joint training mechanisms and talent development by influencing power negotiation and cooperation rules? Thus, a clear academic gap emerges. There is a lack of systematic theoretical and empirical research applying resource dependence theory to analyze the logic of organizational interactions and the dynamics of resource allocation at the micro level in the implementation of Guangdong–Hong Kong–Macao joint training programs. Accordingly, this study applies resource dependence theory and employs systematic qualitative case studies to conduct an in-depth empirical investigation and theoretical development addressing the above issues.

## 3. Theoretical framework

### 3.1. Resource dependence theory: core concepts and subjects of interaction

Resource Dependence Theory (RDT) was systematically introduced by Pfeffer and Salancik in 1978 in External Control of Organisations, with the central proposition that organisations must secure key resources through external interactions to survive and thrive [25]. Resource scarcity shapes the dependency relationships among organisations, affecting their power structures, strategic behaviours and cooperation patterns. In this study, resources are operationalised into four specific categories: policy resources cover program eligibility, financial allocations, and policy authorisation; human resources include instructors, administrators, and managerial staff; knowledge resources relate to curricula, academic norms, and research results; and material resources point to experimental platforms, teaching venues, and research equipment.

Based on the asymmetric nature of resource dependence, dependency structures can be classified into three typical types [26,27]. Symbiotic dependence occurs when two parties possess complementary resources and reciprocal power, as in the balanced distribution of policy resources between regions or the establishment of mechanisms for mutual academic appointments. Dominant dependence arises when one party controls core resources and the other is highly dependent, as in the domination of curriculum standards or research directions by universities in Hong Kong and Macao. Competitive dependence emerges when multiple parties compete for the same scarce resources, such as quotas for special programs or access to experimental facilities among several universities. These resource types and dependency structures form an analytical matrix (see Table 1) that provides a theoretical tool for analysing cooperation networks among universities in Guangdong, Hong Kong, and Macao.

**Table 1:** Multidimensional analysis matrix for resource type-dependency type.

Resource Type/ Dependency	Symbiotic Dependency	Dominant Dependency	Competitive Dependency
Policy Resources	Balanced inter-regional quota allocation; joint application platforms	Key local institutions control quota distribution	Multiple institutions are competing for special program quotas
Human Resources	Mutual recognition of dual supervisors; cross-appointment mechanisms	Hong Kong/Macao supervisors lead curriculum and research	Mainland institutions competing for collaboration with elite mentors
Knowledge Resources	Co-developed curricula; shared data platforms	Hong Kong/Macao institutions export standards and frameworks	Mainland institutions competitively emulating curricula and methodologies
Material Resources	Co-established platforms; shared facilities	Mainland institutions provide infrastructure, while Hong Kong/Macao lead research.	Multiple institutions vying for access to shared experimental facilities

**Fig 1:**
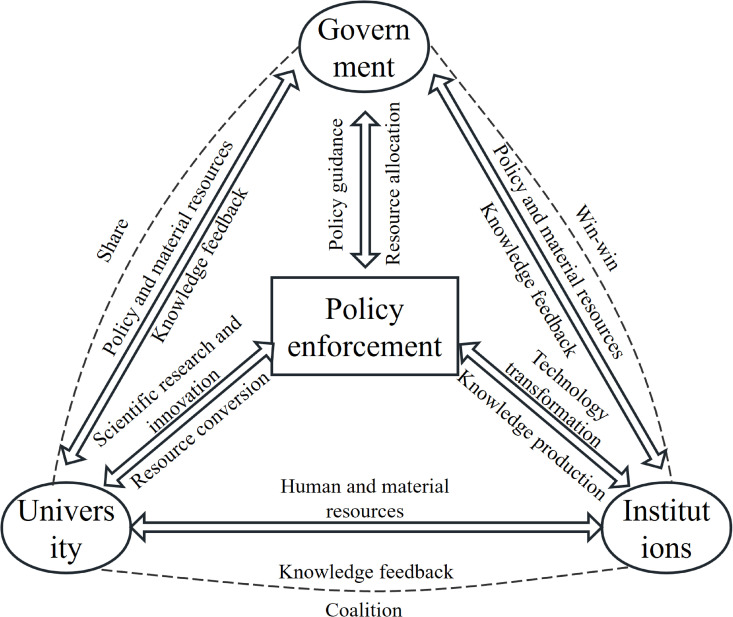
Model of the elements of the policy implementation process.

Three main types of core actors can be identified within the policy implementation network of Guangdong–Hong Kong–Macao joint cultivation. The government provides institutional and financial resources. Universities at key nodes in the resource dependency structure rely on government authorisation to obtain training qualifications. In horizontal cooperation with research institutions, they employ mechanisms such as “curriculum sharing,” “dual tutorship,” and “platform co-construction” to export their advantageous resources, thereby achieving resource complementarity or enhancing their status. Research institutions, in turn, engage in complementary cooperation with universities by responding to industrial demands and providing practice platforms [28]. These three types of actors engage in complex interactions, exchanges, and strategic games surrounding the four categories of resources, as illustrated in Fig 1. The Fig illustrates that the government influences universities and research institutions through policy guidance and resource allocation, while these institutions provide feedback via knowledge production and technology transfer. In addition, alliances and resource exchanges occur among them.

### 3.2. Intermediary mechanisms for policy implementation

The implementation outcomes of the Guangdong–Hong Kong–Macao joint training policy are primarily achieved through two core intermediary mechanisms—resource integration and collaborative governance, which contribute to regional talent development. The resource integration mechanism refers to the effective allocation and coordinated utilisation of key resources from diverse sources and categories through policy implementation, manifested at two levels. First, integrating financial resources aims to enhance infrastructure and expand the scale of postgraduate training through special government funding and local matching funds. Second, integrating human and knowledge resources leverages the complementary strengths of high-level faculty in Hong Kong and Macao and the mainland’s student base through dual-mentoring systems, joint courses, and other collaborative formats. The theoretical foundation of this mechanism lies in Resource-Based Theory [29], which emphasises the integration and utilisation of scarce resources by organisations to gain a competitive advantage.

The collaborative governance mechanism, by contrast, emphasises resource sharing and the complementarity of strengths through the construction of cross-organisational cooperation networks to enhance overall system effectiveness. In joint cultivation projects, this mechanism operates at two levels. First, institutional synergy occurs when the governments and universities of the three regions jointly establish standards—such as cross-border quality assurance and mutual recognition of credits and degrees—to reduce institutional friction and transaction costs. Second, organisational synergy involves the creation of physical platforms, such as university consortia and joint laboratories, to facilitate the substantive flow of talent and resources within the region. The design of such mechanisms aligns with network governance theory’s account [18] of the roles of trust, reputation, and reciprocity in reducing transaction costs and maximising resource utilisation.

### 3.3. Analytical framework model

**Fig 2:**
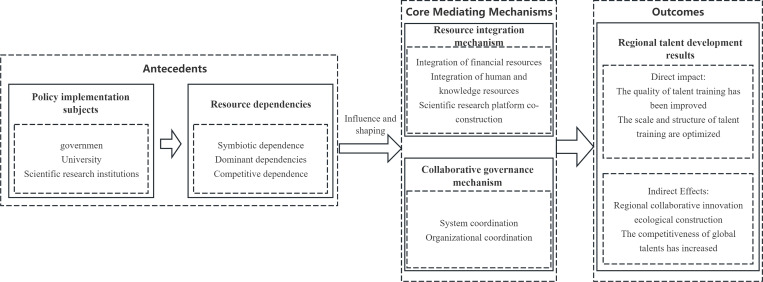
Diagram of the study’s logical framework.

To systematically reveal the pathways through which policy implementation influences talent development, this study builds, based on the above theoretical foundations, an integrated analytical framework of “subject–dependence–mechanism–effectiveness,” the complete logic of which is depicted in Fig 2. The logical starting point of the framework is the core participants in policy implementation. Drawing on Resource Dependence Theory’s focus on inter-organisational interactions, this study identifies three core organisational actors within the network: the government, as the initial allocator of policy and financial resources; universities, as the central implementing units of talent cultivation; and research institutes, as key platforms linking scientific research with industry. Together, they constitute the core nodes of the resource exchange and dependency network. In addition, students—while not organisational actors—are the direct target group of the policy and the ultimate bearers of its effectiveness, and are therefore included at the endpoint of the framework.

Two intermediary mechanisms at the framework’s core bridge actor behaviours and outcomes. Dependencies between actors do not directly produce effectiveness; instead, they operate through “resource integration” and “collaborative governance” mechanisms. Specifically, the resource integration mechanism captures the dynamic process of resource exchange and complementarity among the three organisational actors. In contrast, the collaborative governance mechanism refers to the rules and structures that govern interactions among the parties. As the direct training recipients, students are the ultimate beneficiaries of both mechanisms. For example, they gain access to high-quality faculty and experimental platforms through resource integration and benefit from joint degrees and mutual recognition of credits established via collaborative governance.

The endpoint of the framework is the effectiveness of regional talent development. Effectiveness is reflected in a twofold impact pathway: (1) a direct impact—observable in improvements to the quality and scale of postgraduate training—and (2) an indirect impact—seen in the long-term contribution to building a regional collaborative innovation ecosystem by facilitating knowledge mobility and integrating industry, universities, and research.

## 4. Research methodology

The qualitative case study methodology, which seeks to explore the complex “how” and “why” questions of particular social phenomena in real-life contexts [30], is well aligned with the exploratory objectives of this study. To achieve these objectives, the study adopts two complementary strategies: an embedded case strategy to examine the internal operational logic of a single case, and a cross-case comparison strategy to identify general patterns and key differences across models. Combining these strategies ensures that the analysis balances the depth of micro-level detail with the breadth of macro-level comparison, thereby providing a solid foundation for the validity and credibility of the study’s conclusions.

### 4.1. Research design

The study’s overall design follows a systematic three-step process. First, the case base was constructed, and data were collected by identifying cases through official channels and screening them against predefined criteria, yielding a sample of 47 joint training programs. Second, each case underwent in-depth description and coding, with “thick” descriptions covering its collaborative context, resource allocation model, and governance structure, followed by systematic coding based on the theoretical framework (see Appendices 1 and 2). Third, cross-case model comparison and mechanism refinement were conducted. A systematic comparison of cases with different types of resource dependency identified key causal mechanisms explaining how policy implementation influences talent development, thereby advancing theory building. The study further examines differences in implementation mechanisms and effectiveness between “university–university” and “university–research institute” cases, based on organisational type, to deepen the comparative analysis.

### 4.2. Case selection and data sources

Case selection followed the principle of “theoretical sampling” [31], which seeks to maximise diversity in key theoretical constructs (e.g., types of resource dependence, subject composition) rather than statistical representativeness. First, the initial pool of cases was identified. A search of official websites, policy documents, annual reports, and mainstream media reports from the Guangdong Provincial Department of Education, municipal education bureaus, key universities, and research institutes for the period 2016–2024 identified more than 60 collaborative projects or programs related to “Guangdong–Hong Kong–Macao joint postgraduate training.” Second, inclusion and exclusion criteria were applied: (1) Substantiality of cooperation, projects must have entered substantive operation with student participation, rather than remaining at the framework agreement stage; (2) Accessibility of information, core details, such as the cooperation model and main participants, must be publicly available and cross-validated; (3) Timeframe, projects must have been initiated or active between 2016 and 2024. Third, the final sample was determined. After screening, 47 joint cultivation entities—34 universities and 13 research institutes—were selected as the core analysis sample (see Appendices 1 and 2).

**Fig 3:**
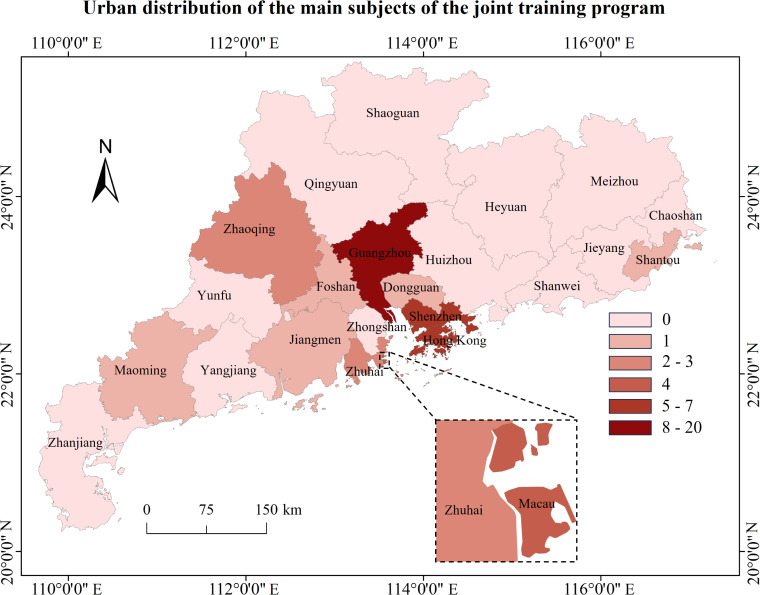
Urban distribution of the main subjects of the joint training program.

Geographically, the sample exhibits a high degree of spatial concentration. As shown in Fig 3, three core cities—Guangzhou, Shenzhen, and Hong Kong—host 33 of the 47 cooperative entities, accounting for over 70% of the total. Guangzhou serves as the dominant core node, with 20 institutions, whereas most cities have only a few participating institutions. This pronounced geographic imbalance reflects the structural reality that higher education and research resources in the Greater Bay Area are concentrated in central cities, providing an important contextual backdrop for the study’s application of resource dependence theory. All data used in this study come from publicly accessible secondary sources, including official government policy documents, university and research institute announcements, program enrollment brochures, annual reports, mainstream news media, and relevant academic research.

The data sources for this study are all publicly accessible secondary sources, including, but not limited to, official government policy documents, university and research institute announcements on official websites, program enrollment brochures, annual work reports, mainstream news media reports, and relevant academic research papers.

### 4.3. Data analysis and coding strategies

The core of the data analysis lies in the systematic coding and categorisation of the 47 cases, with particular emphasis on defining the core variable “resource dependence.” Drawing on resource dependence theory, this study develops coding rules based on two dimensions: “resource complementarity” (whether the parties’ resources are heterogeneous and complementary) and “power asymmetry” (whether one party possesses core resources and dominates decision-making) [26], as shown in Table 2. The 47 cases were independently coded using these rules, and the results were cross-checked. Any inconsistencies were resolved through discussion and by referring to the sources to ensure coding reliability. Following independent coding, the study employed cross-case analysis to distil core findings. Cross-case analysis is a qualitative method that identifies universal patterns and theoretical relationships transcending individual contexts by systematically comparing multiple cases [32]. This method was chosen because it mitigates the limitations of single-case studies and enhances the credibility and generalizability of findings by comparing cases with different dependency structures and collaboration types.

**Table 2:** Resource dependency coding rules and judgment basis.

Dependency Type	Definition	Key Diagnostic Indicators
Symbiotic Dependency	Collaborative partners possess heterogeneous key resources requiring mutual exchange, characterised by relatively balanced and reciprocal power dynamics.	High resource complementarity; Bilateral negotiation and co-management mechanisms (e.g., dual-supervisor systems, joint steering committees); Shared outcomes and risk allocation.
Dominant Dependency	One party controls core scarce resources, creating significant power asymmetry, while the other operates in a dependent, subordinate position.	Unidirectional resource flow; Decision-making is dominated by the controlling entity, with the dependent party primarily executing directives. The dominant actor retains core intellectual property or outcomes.
Competitive Dependency	Multiple homogeneous entities compete for partnership opportunities with the same advantaged institution.	Singular, scarce collaboration objectives; Multiple potential partners vying for identical resources; Primarily manifests during the project initiation and establishment phases.

The analytical process comprised three steps: (1) preparing a “case summary” for each case, detailing key variables; (2) integrating all summaries into a “case–variable” matrix for structured data presentation; and (3) conducting cross-sectional analysis of the matrix data. In this third step, repeated horizontal (across cases) and vertical (within cases) comparisons in the matrix were used to systematically examine and verify theoretical propositions. Such as whether specific dependency structures are associated with particular implementation mechanisms, culminating in the core findings presented in Section 4.

## 5. Findings and discussion

Grounded in the analytical logic of “antecedents–mechanisms–results,” this Section takes resource dependence as the point of departure. It first examines how dependency structures shape the roles of key actors, then analyses how these structures operate through the intermediary mechanisms of resource integration and collaborative governance, and finally assesses the differentiated effects on regional talent development.

### 5.1. Structural antecedents: subject roles and dependencies

Analysis of the 47 joint training cases reveals three core resource dependencies within the policy implementation network. These dependencies not only determine the role configurations and behavioural patterns of the government, universities, and research institutes, but also evolve dynamically as policies develop.

#### 5.1.1. Symbiotic dependence: reciprocal cooperation based on complementary resources.

Symbiotic dependence represents the most dominant relational pattern, observed in 44 out of 47 cases (approximately 94%). It is characterised by stable, reciprocal cooperation between partners with equal status and highly complementary key resources. This structure has distinct role implications for universities and research institutes. In university–university cooperation, mainland universities typically serve as providers of student cohorts and institutionalised teaching systems. In contrast, Hong Kong and Macao universities serve as conduits for internationalised faculty and global research networks. For instance, the partnership between Shenzhen University and the City University of Hong Kong has established a dual-mentorship system that integrates “international academic guidance” with “localised practical training,” resulting in the steady cultivation of over 100 master’s and doctoral students within five years [33]. In the newer university–research institute symbiotic model—emerging during the policy deepening phase—the division of roles is even more explicit. Universities are responsible for systematic theoretical instruction and degree conferment, while research institutes such as the Southern Oceanic Laboratory contribute cutting-edge research agendas and advanced experimental infrastructure. Notably, the Hong Kong–Shenzhen patent cooperation rate in this context has reached 68.03% [34], underscoring the Institute’s central role as both an industrial technology integrator and an innovation practice platform.

#### 5.1.2. Dominant versus competitive dependence: power games in asymmetric structures.

When key resources are highly scarce and unevenly distributed, dominant and competitive dependency structures emerge, producing unequal power relations among actors. Although dominant dependence appears in only 3 cases (6%), it clearly shows how top-tier institutions function as rule-setters. The establishment of the Hong Kong University of Science and Technology (Guangzhou) illustrates this dynamic: as a branch campus, its curriculum design, academic standards, and institutional reputation flow unidirectionally from the parent university, while the mainland partner assumes the role of resource provider, offering land, funding, and administrative support. Similarly, in the collaboration between the Guangdong–Hong Kong–Macao Greater Bay Area Institute of Precision Medicine and Fudan University, all aspects of academic status and core curriculum remain under Fudan’s control, with the Institute serving primarily as a research and practice base. In such cases, pronounced power asymmetry dictates a one-way allocation of resources.

**Fig 4:**
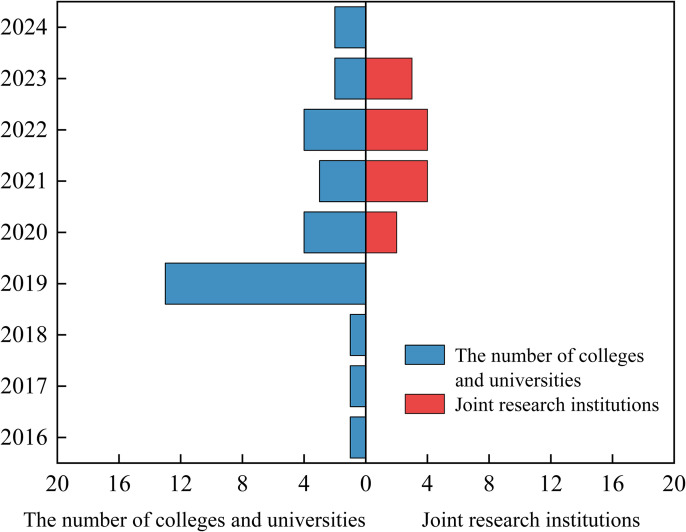
Trend of temporal distribution of cooperative subjects in Guangdong-Hong Kong-Macao joint training programs.

Competitive dependence, by contrast, has intensified since the government repositioned itself as the central allocator of core resources. Following 2019 (see Fig 4), Guangdong Province allocated RMB 270 million in special funding for joint training programs (Nanfang Daily News 2024), sharply increasing competition among local institutions. These actors, now functioning as active seekers, compete vigorously for limited program quotas and subsidies, particularly to secure partnerships with high-quality resources from Hong Kong and Macao. Such rivalry underscores how state-led resource centralisation reshapes horizontal competition within the policy network.

### 5.2. Core intermediary mechanisms for policy implementation

#### 5.2.1. Mechanisms for resource integration: the clustering effect of financial, human, and scientific platforms.

Resource integration reflects the capacity to mobilise and combine critical elements such as funding, faculty, and research infrastructure. In financial allocation, a distinct concentration pattern is evident. For instance, under the Guangdong–Hong Kong–Macao Science and Technology Innovation Joint Funding Scheme, South China Normal University, Guangdong University of Technology, and South China Agricultural University collectively received over 30% of the total funding [35]. While enhancing project capacity at leading institutions, such concentration risks producing a “Matthew effect” that may undermine balanced regional development.

Integrating scientific research resources is particularly prominent in university–institute collaborations, where complementary functions amplify synergy. The partnership between the Southern Ocean Laboratory and the Hong Kong University of Science and Technology has resulted in a joint deep-sea research platform, the training of 10 doctoral candidates, and the publication of 15 high-impact papers within three years. In contrast, university–university collaborations emphasise the alignment of teaching resources and human capital. Data show that research institutes are more likely than universities to lead or co-lead projects involving “dual mentorship” models, reflecting their deeper engagement in joint supervision arrangements [36].

#### 5.2.2. Co-governance mechanisms: governance logic of institutional interface and organisational linkages.

Collaborative governance ensures the sustained operation of projects through coordinated institutional arrangements and cross-organisational cooperation. Governance structures vary by collaboration mode. In university–institute cooperation, dedicated management bodies are more common. For example, the joint laboratory established by HKUST and Guangzhou University operates under an independent project management committee, with co-managed financial oversight and joint evaluation of outputs, enabling greater operational efficiency. Conversely, university–university collaborations tend to adopt flatter governance structures, relying on interdepartmental agreements and faculty-led initiatives. In the cooperation between South China Agricultural University and the City University of Macau, although curriculum alignment and mutual degree recognition have been achieved, the absence of a unified quality assessment framework has introduced the potential risk of excessive implementation flexibility [37].

### 5.3. Pathways of policy implementation to regional talent development

The intermediary mechanisms outlined above transmit the effects of resource dependencies into tangible outcomes for regional talent development. Drawing on logic modelling in policy evaluation, the influence pathways can be divided into direct impacts—those observable in immediate policy targets such as postgraduate programs and individual students—and indirect impacts—longer-term contributions to the regional innovation ecosystem.

#### 5.3.1. Direct impact: quality improvement and structural optimisation of human resources training.

Policy mechanisms have directly improved postgraduate training quality and optimised the scale–structure balance at both the institutional design and organisational implementation levels. Mechanism innovations such as dual mentorship systems and credit mutual recognition have expanded the content dimensions of training, enabling students to access integrated learning experiences that significantly strengthen research competencies. The Redbird Cross-Campus Program, integrating resources from HKUST and its Guangzhou campus, served over 1,300 postgraduates between 2020 and 2024, with graduates demonstrating higher research-to-application conversion rates than peers in single-system programs. Moreover, policy execution has facilitated the coordination of regional education resources. By 2024, diversified training modes—from dual tutorship and joint degrees to university–enterprise practicum—had shifted the regional talent supply model from fragmented provision to systematic integration [38,39].

#### 5.3.2. Indirect impacts: building a regional collaborative innovation ecosystem.

Beyond immediate educational outcomes, the policy has contributed to a regional high-end talent ecosystem by strengthening organisational networks and fostering systemic linkages. Joint training has accelerated the integration of research, education, and industry. A notable example is the collaboration among Guangzhou Pharmaceutical Group, Guangzhou Medical University, and the Macao University of Science and Technology, which established a respiratory health research platform aligned with joint doctoral candidate training. Around the Panlangen antiviral project, this collaboration completed the full cycle from research initiation and postgraduate training to patent registration and product commercialisation, producing over 20 academic papers, securing 15 invention patents, and achieving market delivery within three years. This illustrates how coordinated project design and resource matching enhance the region’s capacity for technology transfer. The mechanisms also support international talent attraction. The Shenzhen Bay Laboratory, working with HKUST, launched the Global Top Scholars Introduction Program, bringing in more than 100 internationally recognised doctoral supervisors by 2024. Through dual-mentorship arrangements and co-built research platforms, the program has formed a closed loop of “attraction–training–retention,” reinforcing the Greater Bay Area’s position in the global high-end talent market [40,41].

### 5.4. Policy implications for optimising joint cultivation governance

Based on the findings, this study proposes systematic reforms in platform construction and institutional innovation to enhance resource sharing, leverage complementary advantages, and maximise the policy effectiveness of the GBA joint cultivation program.

First, addressing the structural imbalance in resource allocation is crucial, and this can be achieved by building an open, shared, collaborative ecosystem. High-quality cooperation opportunities and financial resources are currently concentrated in a small number of elite institutions. Policies should therefore move beyond simple project funding toward establishing performance-based structural subsidies that activate the collaborative potential of the entire region. Furthermore, the governments of the three regions should jointly establish a permanent “Bay Area Education Resource Sharing Platform” to pool and openly share key resources, including a supervisor database, course modules, and a directory of laboratory equipment. This approach can draw on the experience of the University of California (UC) system [42], which uses a unified digital library and course exchange mechanism to facilitate resource complementarity between its top-tier and other campuses, thereby enhancing system-wide effectiveness and offering a valuable model for promoting regional educational equity and quality. A corresponding “resource sharing incentive fund” should be created to motivate participation in this platform to provide financial awards to institutions that open their resources and generate positive synergies, activating underutilised educational assets throughout the region.

Second, the institutionalisation of cooperation models must be promoted to guarantee the Sustainability of collaborative relationships. Most collaborations rely on ad hoc, non-institutionalised agreements lacking long-term stability. The EU’s Bologna Process provides a valuable blueprint for the transition from a project-driven to an institution-driven approach [43]. A permanent “Bay Area Higher Education Coordinating Body” should be established based on its core concepts. Its responsibilities would include coordinating management and promoting a unified, internationally aligned system for cross-border quality assurance and credit recognition, similar to the European Credit Transfer System (ECTS). Strong institutional frameworks are the only way to provide a solid foundation for in-depth cooperation. Finally, a lifecycle tracking and feedback system for talent cultivation should be established. By monitoring the long-term development of graduates, such a system would provide empirical evidence for dynamic policy adjustments, creating a self-improving governance loop.

## 6. Conclusions

### 6.1. Conclusions of the study

Based on resource dependence and collaborative governance theories, this study examines the policy implementation of the Guangdong-Hong Kong-Macao joint cultivation program. It constructs an analytical path of “resource structure - implementation mechanism- talent effectiveness” to systematically investigate the resource allocation and collaboration mechanisms among the government, universities, and research institutes. The study finds that the program promotes resource integration through financial support, talent recruitment, and platform co-construction. This has led to an institutionalised mechanism centred on dual-supervisor systems, credit recognition, and enterprise-based practical training, significantly enhancing postgraduate students’ research abilities and cross-border adaptability. Furthermore, establishing joint laboratories and inter-university research platforms has strengthened regional and extra-regional scientific networks, indirectly enhancing the Greater Bay Area’s international talent attraction and competitiveness. A comparison of “university-university” and “university-research institute” cooperation models reveals significant differences in the scale, output, and regional mobility of talent cultivation, contingent on varying resource structures and implementation mechanisms. The study further argues that policy implementation mechanisms mediate the relationship between institutional design and regional talent development. The rationality of resource allocation and collaborative structures is therefore key to attracting high-level regional talent and fostering educational innovation.

### 6.2. Research limitations and future prospects

While this study offers a theoretical contribution, several limitations should be noted. First, the reliance on secondary data, such as public policy texts and official reports, provides a macro-level overview but may not fully capture the informal negotiations and power dynamics inherent in inter-organisational partnerships. Second, because competitive processes are covert, the classification of “competitive dependence” relies on indirect evidence and qualitative judgment; its specific mechanisms of influence thus require further investigation. In light of these limitations, future research could employ in-depth fieldwork, such as interviews with key policymakers and university administrators, to reveal the underlying motivations and barriers to cooperation.

## Supporting information

S1 Appendix List of Cooperating Universities of Guangdong, Hong Kong and Macao for Joint Graduate Programs. A list of participating higher education institutions in the Greater Bay Area.
(DOCX)

S2 Appendix Status of Guangdong-Hong Kong-Macao Joint Postgraduate Training at HKITAR. Summary data on Training Model Description, Collaboration Commencement, Partner University/Universities and Dependency Type for the collaborative training initiative.
(DOCX)
