# Supplementary material for: Study on the policy implementation of the Guangdong-Hong Kong-Macao joint graduate training program and regional talent development
Source: PLoS One. 2025 Dec 29;20(12):e0338940. doi: 10.1371/journal.pone.0338940 (PMC12747370; doi:10.1371/journal.pone.0338940)
Supplement: S1 Appendix — A list of participating higher education institutions in the Greater Bay Area. (DOCX) [file pone.0338940.s001.docx]

**S1 Appendix List of Cooperating Universities of Guangdong, Hong Kong and Macao for Joint Graduate Programs**

| **Region** | **University** | **Participation Year** | **Collaborative Training Model Description** | **Dependency Type** | **Classification Rationale** |
| --- | --- | --- | --- | --- | --- |
| **Guangdong** |  |  |  |  |  |
|  | Sun Yat-sen University | 2017 | Dual-supervisor PhD system + research funding; industry-academia-research collaboration platform (Dongguan Institute) | Symbiotic Dependency | High resource complementarity (dual supervision, tripartite collaboration), equal control rights, no dominant party |
|  | South China University of Technology | 2022 | GAC R&D Center joint project contracting system: "Industry problem-setting → academic solution → joint evaluation" closed-loop | Symbiotic Dependency | Resource complementarity (industry-academia partnership), shared governance (closed-loop mechanism), balanced partnership |
|  | South China Normal University | 2020 | Joint educational psychology laboratory; "dual-campus + dual-supervisor" system | Symbiotic Dependency | High resource complementarity (shared facilities/mentors), low control disparity (balanced campus roles) |
|  | Jinan University | 2019 | Overseas Chinese studies program; Macau-Zhuhai cross-border fieldwork base | Symbiotic Dependency | Resource complementarity (cross-border facilities), equal authority (joint research), Macau partnership |
|  | Guangdong University of Foreign Studies | 2021 | Legal translation talent centre; collaboration with Hong Kong/Macao courts on case translation | Symbiotic Dependency | Resource complementarity (talent-court cooperation), balanced control, no dominant entity |
|  | Guangzhou University of Chinese Medicine | 2020 | Clinical rotation at the Macau Health Bureau; joint Chinese medicine standardisation laboratory | Symbiotic Dependency | High resource complementarity (clinical/lab facilities), shared governance, Macau involvement |
|  | Southern Medical University | 2023 | Medical big data platform; joint medical case repository with Hong Kong Hospital Authority | Symbiotic Dependency | Resource complementarity (co-built data infrastructure), balanced authority (joint development), Hong Kong linkage |
|  | Guangdong University of Technology | 2024 | Huawei "Innovation Workshop"; smart manufacturing talent development pipeline | Symbiotic Dependency | Resource complementarity (industry-academic training chain), shared control, no dominance |
|  | Wuyi University | 2020 | Hong Kong PolyU base + Jiangmen industrial park practicum platform | Symbiotic Dependency | High resource complementarity (shared facilities), equal control rights, Hong Kong PolyU partnership |
|  | Shantou University | 2022 | Marine biotechnology joint laboratory; "Shantou + Hong Kong" segmented training | Symbiotic Dependency | Resource complementarity (shared lab/phased training), balanced control, Hong Kong academic involvement |
|  | Shenzhen University | 2021 | Cross-border joint master/doctoral program operating through Hong Kong campus | Symbiotic Dependency | Resource complementarity (cross-border training), balanced authority (collaborative program), no dominant party |
|  | Guangdong Polytechnic Normal University | 2021 | Dedicated funding mechanism for graduate student exchanges with Hong Kong/Macao | Symbiotic Dependency | Resource complementarity (exchange support), equal control rights, Hong Kong/Macao involvement |
|  | Guangdong University of Finance and Economics | 2022 | Domestic-international academic system; cross-border curriculum modules | Symbiotic Dependency | Complementary academic systems, shared courses, balanced authority |
|  | Beijing Normal University (Zhuhai) | 2019 | "Academic problem-setting → local application" practicum platform | Symbiotic Dependency | Complementary educational resources, applied local focus, shared governance |
|  | Foshan University | 2019 | Engineering "industry-academic dual supervision"; smart manufacturing training base | Symbiotic Dependency | Strong industry participation, complementary mentorship, balanced control |
|  | Zhaoqing University | 2019 | Joint education administration training; course credit reciprocity, and faculty sharing | Symbiotic Dependency | Complementary educational resources, balanced authority, and no dominant party |
|  | Zhuhai College of Science and Technology | 2019 | IT field project-based teaching; joint practicum platform with local tech firms | Symbiotic Dependency | Project-driven pedagogy, shared corporate resources, equal governance |
|  | Guangdong Petrochemical University | 2019 | Integrated "research + internship" mechanism for energy/chemical engineering | Symbiotic Dependency | Combined research-practice approach, resource complementarity, shared control |
|  | Guangzhou Maritime University | 2019 | Shipping engineering "industry mentor + academic supervisor" dual guidance | Symbiotic Dependency | Dual-supervisor system, shared industry resources, balanced authority |
|  | Guangzhou Nanfang College | 2019 | Business "corporate case studies + joint research projects" | Symbiotic Dependency | Complementary pedagogy-research integration, shared governance |
|  | Guangdong University of Education | 2019 | Education discipline "teaching practicum + research collaboration" dual-track model | Symbiotic Dependency | Teaching-resource complementarity, shared control |
|  | Guangzhou City University of Technology | 2019 | Engineering "project incubation + industry mentors" mechanism | Symbiotic Dependency | Complementary incubation resources, shared mentorship, equal authority |
|  | Guangdong Institute of Science and Technology | 2019 | STEM "research platform + corporate training" collaboration; joint laboratories | Symbiotic Dependency | Platform-practicum resource synergy, shared governance |
|  | Guangdong University of Finance | 2019 | Fintech "industry problem-setting + academic solution" framework; joint research fund | Symbiotic Dependency | Complementary problem-solving mechanism, co-established funding, balanced control |
| **Hong Kong** |  |  |  |  |  |
|  | University of Hong Kong | 2018 | Financial risk specialisation; cross-border Shenzhen-Hong Kong simulated trading system | Symbiotic Dependency | High resource complementarity (cross-border platform), shared governance, Shenzhen partnership |
|  | Chinese University of Hong Kong | 2016 | Cross-border credit transfer system; joint fintech projects | Symbiotic Dependency | Resource complementarity (credit reciprocity/joint projects), equal control, Guangdong involvement |
|  | Hong Kong University of Science and Technology | 2019 | AI + Fintech interdisciplinary pilot at the Guangzhou campus | **Dominant Dependency** | Limited resource complementarity (campus homogeneity), high control disparity (HKUST-led experimentation) |
|  | Hong Kong Polytechnic University | 2019 | Rail transit "tri-location rotational training"; Hong Kong-Zhuhai-Macao Bridge case studies | Symbiotic Dependency | High resource complementarity (multi-location rotation), balanced authority, Guangdong/Macao involvement |
|  | Hong Kong Baptist University | 2020 | Joint data science program with BNU (UIC platform in Zhuhai) | Symbiotic Dependency | Resource complementarity (joint program), balanced control (UIC in Zhuhai), Guangdong linkage |
|  | Lingnan University | 2021 | Social Policy Research Centre; Cross-border livelihood data collection | Symbiotic Dependency | Resource complementarity (data collection), shared governance, Guangdong cooperation |
| **Macao** |  |  |  |  |  |
|  | University of Macau | 2019 | Lusophone culture curriculum + comparative law research base | Symbiotic Dependency | Resource complementarity (academic programs/research facility), balanced control, potential collaborations |
|  | Macau University of Science and Technology | 2022 | Traditional Chinese Medicine Quality Evaluation Centre; standardisation laboratory | Symbiotic Dependency | High resource complementarity (evaluation centre/lab), equal authority, potential partnerships |
|  | City University of Macau | 2023 | Tourism management "tri-location practicum rotation"; collaboration with Disney/Chimelong | Symbiotic Dependency | Resource complementarity (practicum partnerships), balanced control, Guangdong linkage (Chimelong) |
|  | Macao Polytechnic University | 2024 | Intelligent systems engineering laboratory; "Macao theory + Hengqin practice" model | Symbiotic Dependency | Resource complementarity (theory-practice integration), shared governance, Hengqin (Guangdong) cooperation |
