# Supplementary material for: Study on the policy implementation of the Guangdong-Hong Kong-Macao joint graduate training program and regional talent development
Source: PLoS One. 2025 Dec 29;20(12):e0338940. doi: 10.1371/journal.pone.0338940 (PMC12747370; doi:10.1371/journal.pone.0338940)
Supplement: S2 Appendix — Summary data on Training Model Description, Collaboration Commencement, Partner University/Universities and Dependency Type for the collaborative training initiative. (DOCX) [file pone.0338940.s002.docx]

**S2 Appendix Status of Guangdong-Hong Kong-Macao Joint Postgraduate Training at HKITAR**

| **Research Institution** | **Partner University/Universities** | **Collaboration Commencement** | **Training Model Description** | **Dependency Type** | **Classification Rationale** |
| --- | --- | --- | --- | --- | --- |
| Guangzhou Institute of Respiratory Health | University of Macau, Macau University of Science and Technology | 2020 | One-year exchange program complemented by mutual visits and virtual meetings | Symbiotic Dependency | High resource complementarity (exchange & visits), equal control rights (joint management) |
| Shenzhen Bay Laboratory | Hong Kong University of Science and Technology, University of Hong Kong | 2021 | Years 1 and 4 in Hong Kong; Years 2 and 3 in Shenzhen | Symbiotic Dependency | Resource complementarity (dual-location resources), shared control (time allocation), no dominant party |
| Huawei Technologies Co., Ltd. | The Chinese University of Hong Kong (Shenzhen) | 2021 | A dual-supervisor system integrates university coursework with corporate practice. | Symbiotic Dependency | High resource complementarity (academic & practical training), equal control rights (dual supervisors) |
| Guangdong Institute of Intelligent Science and Technology | University of Macau | 2022 | Dual-mentorship model combining academic courses in Macau with research in Guangdong | Symbiotic Dependency | Resource complementarity (coursework & research), equal control rights (dual supervisors) |
| Dongguan Greater Bay Area Institute of Advanced Study | Chinese University of Hong Kong, Sun Yat-sen University | 2020 | Dual-supervisor system with primary supervision by CUHK mentors; ≥50% time at CUHK campus | **Dominant Dependency** | Limited resource complementarity (CUHK-centric resources), high control disparity (CUHK-led supervision) |
| Shenzhen International Quantum Research Centre | University of Macau, Chinese University of Hong Kong | 2021 | Full-time immersive study under a dual-supervisor framework | Symbiotic Dependency | Resource complementarity (multi-institutional collaboration), equal control rights (dual supervisors) |
| Southern Marine Science and Engineering Guangdong Laboratory (Zhuhai) | University of Macau | 2022 | Year 1: Academic study in Macau; Years 2-4: Research practice | Symbiotic Dependency | High resource complementarity (academic & research phases), shared control (time allocation) |
| Shenzhen Institute of Advanced Technology (SIAT), CAS | University of Macau, Hong Kong Polytechnic University | 2023 | Dual-supervisor system: Year 1 in Macau; Years 2-3 conducting research in Shenzhen | Symbiotic Dependency | Resource complementarity (multi-location resources), equal control rights (dual supervisors) |
| IDEA Institute | Hong Kong University of Science and Technology (Guangzhou) | 2023 | Dual-mentorship model: HKUST(GZ) coursework + IDEA research practice | Symbiotic Dependency | Resource complementarity (academic & applied research), shared control (dual supervisors) |
| Greater Bay Area Institute of Precision Medicine (Guangzhou) | Fudan University | 2022 | Student enrollment managed by Fudan; coursework at Fudan; research conducted at the Institute | **Dominant Dependency** | Limited resource complementarity (compartmentalised resources), high control disparity (Fudan dominance in enrollment & academics) |
| Guangdong-Hong Kong-Macao Development Research Centre | Education University of Hong Kong, Sun Yat-sen University | 2021 | Initial focus on joint research and exchanges, exploring future postgraduate training models | Symbiotic Dependency | Resource complementarity (research & scholarly exchange), equal control rights (collaborative exploration) |
| Peng Cheng Laboratory | Sun Yat-sen University | 2022 | Joint cultivation with a dual-supervisor system, emphasising research practice | Symbiotic Dependency | Resource complementarity (theoretical & practical training), shared control (dual supervisors) |
| Guangzhou National Laboratory | Sun Yat-sen University | 2023 | Dual-supervisor model: SYSU coursework + laboratory-based research practice | Symbiotic Dependency | Resource complementarity (academic & experiential learning), shared control (dual supervisors) |
